# Serum sialylation changes in cancer

**DOI:** 10.1007/s10719-018-9820-0

**Published:** 2018-04-21

**Authors:** Zejian Zhang, Manfred Wuhrer, Stephanie Holst

**Affiliations:** 1 Center for Proteomics and Metabolomics, Leiden University Medical Center, Postzone S3, Postbus 9600, 2300 RC Leiden, NL The Netherlands; 2 Department of Biochemistry and Molecular Biology, Key Laboratory of Glycoconjugate Research Ministry of Public Health, School of Basic Medical Sciences, Fudan University, Shanghai, China

**Keywords:** Serum, Sialylation, Cancer biomarker, Glycosylation

## Abstract

Cancer is a major cause of death in both developing and developed countries. Early detection and efficient therapy can greatly enhance survival. Aberrant glycosylation has been recognized to be one of the hallmarks of cancer as glycans participate in many cancer-associated events. Cancer-associated glycosylation changes often involve sialic acids which play important roles in cell-cell interaction, recognition and immunological response. This review aims at giving a comprehensive overview of the literature on changes of sialylation in serum of cancer patients. Furthermore, the methods available to measure serum and plasma sialic acids as well as possible underlying biochemical mechanisms involved in the serum sialylation changes are surveyed. In general, total serum sialylation levels appear to be increased with various malignancies and show a potential for clinical applications, especially for disease monitoring and prognosis. In addition to overall sialic acid levels and the amount of sialic acid per total protein, glycoprofiling of specific cancer-associated glycoproteins, acute phase proteins and immunoglobulins in serum as well as the measurements of sialylation-related enzymes such as sialidases and sialyltransferases have been reported for early detection of cancer, assessing cancer progression and improving prognosis of cancer patients. Moreover, sialic-acid containing glycan antigens such as CA19–9, sialyl Lewis X and sialyl Tn on serum proteins have also displayed their value in cancer diagnosis and management whereby increased levels of these factors positively correlated with metastasis or poor prognosis.

## Introduction

Cancer, an increasing burden worldwide, is a major cause of death in both developing and developed countries. In 2012, about 14.1 million new cancer cases and 8.2 million deaths from cancer are estimated to have occurred worldwide [1, 2]. Understanding the complex cancer biology and employing reliable biomarkers for detecting and staging malignant diseases and for evaluating various therapeutic approaches can facilitate early detection, efficient therapy and prognosis of cancer and may thereby greatly enhance survival [3–5]. Glycosylation is an important and prevalent modification of proteins and lipids, and is involved in numerous key physiological and pathological processes including malignant transformation, cancer progression and metastasis [6–9]. Aberrant glycosylation has been recognized to be one of the hallmarks of cancer as glycans participate in many cancer-associated events such as cell differentiation, migration, adhesion, invasion, metastasis, cell signaling and trafficking [5, 9–13]. Exploiting differences in glycosylation between malignant and healthy individuals therefore offers opportunities to reveal aspects of the complex cancer biology and to identify more sensitive and specific cancer biomarkers.

**Fig. 1:**
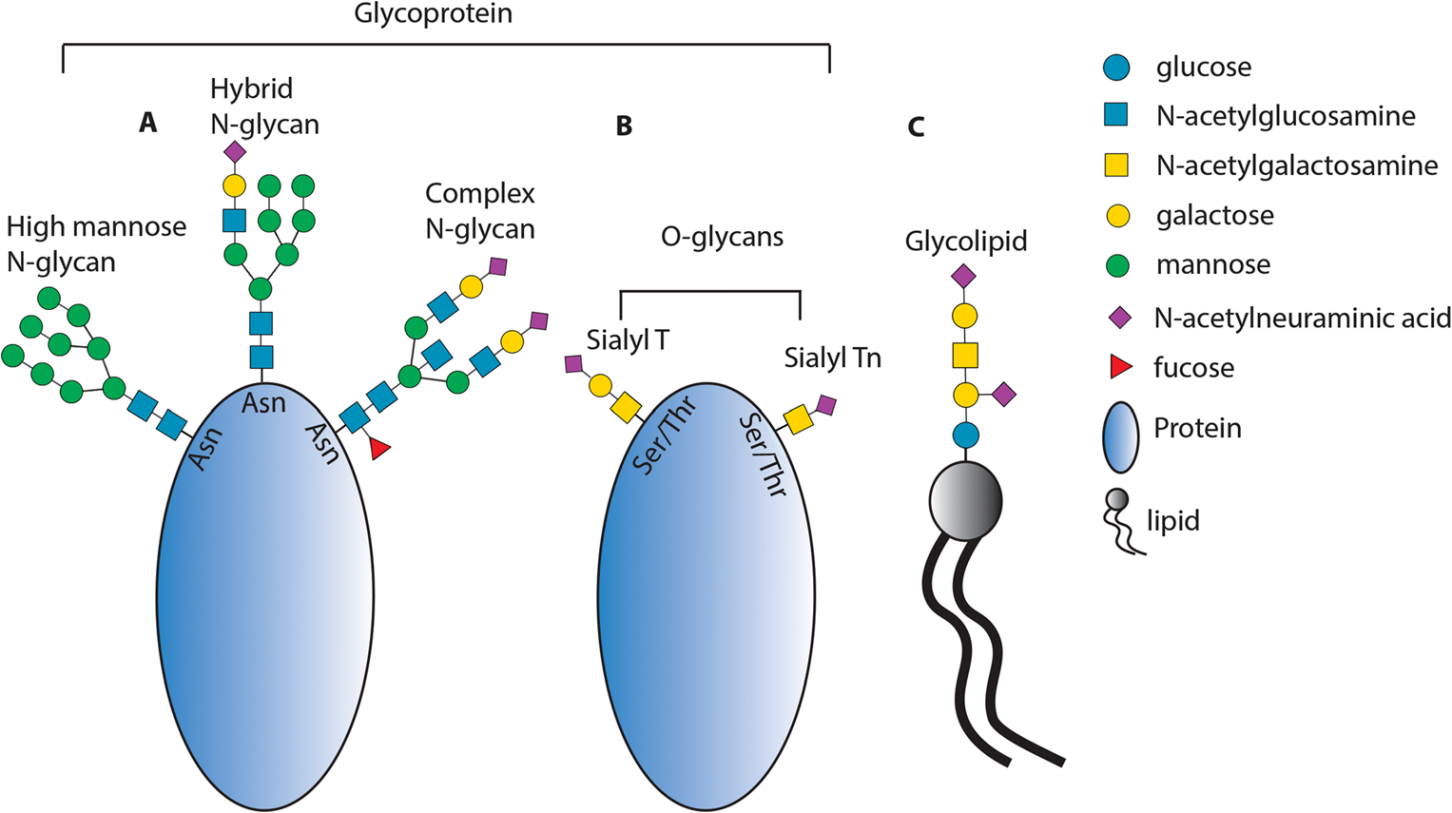
Schematic representation of *N-*linked and O-linked glycans on glycoproteins and glycolipids

The two main types of glycans are *N-*linked and *O*-linked glycans (Fig. 1), which in mammals are composed of the building blocks *N-*acetylglucosamine, galactose, *N-*acetylgalactosamine, fucose, mannose, and sialic acid, and are present on most proteins in human cells and blood circulation [14–16]. Numerous studies have shown that changes in serum/plasma glycan structures occur during cancer initiation, progression, and treatment. This makes glycan markers from serum/plasma a promising, non-invasive group of novel biomarkers for diagnosis, prognosis, and treatment monitoring [10, 17, 18]. Changes in serum *N-* and *O*-linked glycan structures occur not only on cancer-derived cells and proteins, but also on B lymphocyte-derived immunoglobulins and liver-synthesized acute phase proteins such as haptoglobin, α-1-antitrypsin and α-1-acid glycoprotein. This suggests that altered glycosylation may be the result of a systemic tumor response. Therefore, glycans are potentially suitable biomarkers associated with system malfunction in the blood circulation of cancer patients [19–23].

**Fig. 2:**
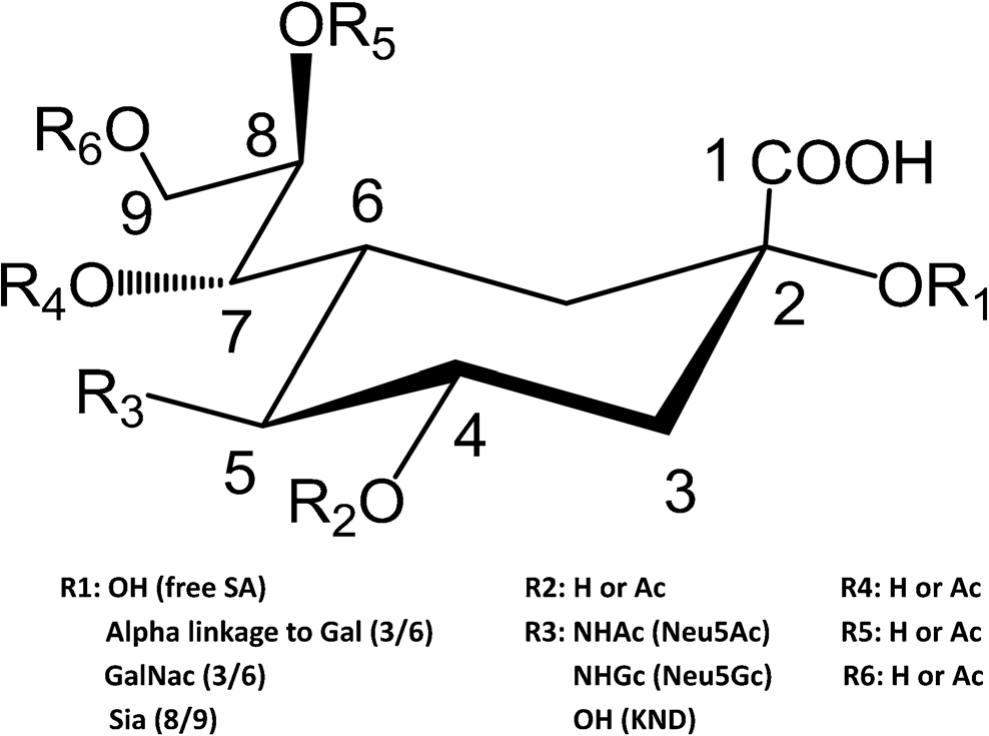
The structures of neuraminic acid and its derivatives

Sialic acids are an important group of monosaccharides with regard to cancer-associated glycan changes. The most abundant forms of this sugar are derivatives of the neuraminic acid, consisting of a nine-carbon backbone, a carboxyl group, and an amino group that is substituted by either an acetyl or glycolyl group (Fig. 2). The most common sialic acid derivative in mammals is *N-*acetylneuraminic acid (Neu5Ac). Another derivative is *N-*glycolylneuraminic acid (Neu5Gc) [24, 25]. However, humans are unable to synthesize Neu5Gc as a consequence of genomic mutations. The biosynthesis of Neu5Gc arises from the action of a hydroxylase that converts the nucleotide donor cytidine monophosphate *N-*acetylneuraminic acid (CMP-Neu5Ac) to cytidine monophosphate *N-*glycolylneuraminic acid (CMP-Neu5Gc). This enzymatic activity is present in animal cells, but not in human cells due to a partial deletion in the gene that encodes CMP-Neu5Ac hydroxylase [26, 27]. For simplicity, the generic term ‘sialic acid’ is used throughout this review when referring to *N-*acetylneuraminic acid unless stated otherwise.

Sialic acids are abundant on various glycoproteins and glycolipids (gangliosides) and are usually terminally attached to the end of the glycan. This forms an outer layer on the cell membrane and glycoconjugates. Sialic acids are linked to other sugars such as galactose or *N-*acetylhexosamine via α2–3- or α2–6-glycosidic bonds or through poly-sialic acid repeats in α2–8-linkage, all catalyzed by specific enzymes. Furthermore, sialic acids exist with different modifications in which the hydroxyl groups may either be methylated or esterified with acetyl, lactyl, phosphate, or sulfate groups (Fig. 2) [28].

As a result of their location, ubiquitous distribution and unique structural features, sialic acids can mediate a wide variety of physiological and pathological processes. They play a role in cellular functions such as transport of positively charged compounds, cellular interaction, conformational changes of glycoproteins on cell membranes, and even masking cell surface antigens [25]. Aberrant sialylation has been implicated in the disturbance of cell-cell recognition, cell adhesion, antigenicity, protein targeting and invasion [29–35]. Studies of malignant cells have revealed alterations in cell surfaces and membranes in terms of the sialic acid content of glycoproteins and glycolipids; accordingly, increased sialylation is one of the main characteristics of malignant transformation [36–42]. Notably, the glycoproteins and glycolipids expressed by tumors can be released into the serum through increased turnover, secretion, and/or shedding [43, 44].

The documentation of alterations in the turnover and release of tumor cell surface glycoconjugates have stimulated investigators’ interest in the measurement and evaluation of serum/plasma sialoglycoproteins and sialoglycolipids. Several investigators have studied their levels including different forms of sialic acids in the serum or plasma of patients with malignant disease. Unlike tumor antigens, which are associated with a limited spectrum of tumors, increased sialic acid levels appear to be a common phenomenon of a variety of neoplastic cells and have been traced down to enhanced sialyltransferase activity, reduced sialidase activity and/or increased sialylglycoprotein production [25, 45]. As a result, monitoring serum/plasma factors such as overall sialic acid content, sialidase activity and sialyltransferase expression, as well as sialylation changes on specific serum glycoproteins may have useful clinical applications for the detection, staging and prognosis of different diseases and cancers as reviewed in the following. In addition, the methods available to measure serum/plasma sialic acid as well as possible underlying biochemical mechanisms involved in the serum/plasma sialylation changes are discussed.

## Overall serum sialic acid levels as markers for malignancy

The first sialic acid measurements were made by Winzler in 1958, MacBeth and Bekesi in 1962 and continued by Brozmanova 10 years later [46–48]. These studies primarily focused on the overall sialic acid levels as total sialic acid (TSA) content which included glycoprotein- and glycolipid-bound sialic acids, and small amounts of free sialic acid, as well as glycolipid-bound sialic acids (LSA) only. Between 98% and 99.5% of TSA found in serum and plasma is bound to glycoproteins and only a small fraction of the sialic acids is bound to lipids, mostly in the form of gangliosides [31]. Normal serum TSA levels of a healthy individual are in the range of 51 to 84 mg/dl, while the lipid fraction only accounts for 0.4 to 0.9 mg/dl of sialic acids [49]. Increased serum TSA, LSA or normalized sialic acid levels such as TSA/total protein (TP) and bound sialic acids/TP were discovered in different types of cancer and have repeatedly been reported to have potential for cancer diagnosis, staging and prognosis.

Shah *et al.*, for example, evaluated serum TSA by a spectrophotometric method as well as linkage-specific sialylation via lectins in the serum of oral cancer patients and controls. Cancer patients were followed up after initiation of anticancer treatment and the patients’ response to the anticancer treatment was assessed. They found significantly higher serum levels of TSA and TSA/TP in oral pre-cancerous conditions as compared to healthy controls. In addition to higher serum levels of TSA and TSA/TP, specifically α2–6-sialylation was found to be increased in untreated oral cancer patients as compared to healthy controls, oral pre-cancerous conditions and responders, while levels of these markers in non-responders were comparable to that of untreated patients. Furthermore, serum α2–3-sialylation levels of non-responders were higher than those of responders. Overall, these sialylation changes in serum correlated to neoplastic transformation and disease progression [50]. In line with this, Sawhney *et al.* evaluated the usefulness of serum TSA and serum LSA as markers for the early detection and staging of oral cancer by spectrophotometric method. This study confirmed that serum TSA and LSA levels were significantly elevated in oral pre-cancer and cancer patients when compared to healthy controls, and progressively increased with grades of dysplasia in precancerous groups and with the extent of malignant disease (TNM Clinical staging) as well as histopathological grades in the cancer group. Serum LSA, in particular, appeared to show potential clinical utility in predicting premalignant changes [51]. Accordingly, the diagnostic potential of overall serum sialic acids as markers in oral cancer has been reported in several other studies [52–57]. Furthermore, total serum sialic acid levels have been shown to alter in ovarian cancer [58], cholangiocarcinoma [59], cervical cancer [60], leukemia [61], colorectal cancer [62, 63], breast cancer [64] and lung cancer [65] which has pointed towards their clinical usefulness as a potential diagnostic and/or prognostic tumor marker. Similarly, Dwivedi *et al.* showed that plasma LSA could be useful as a prognostic determinant in a variety of neoplastic conditions (breast cancer, lung cancer, colon cancer, ovarian cancer, prostate cancer, leukemia, gastrointestinal, thyroid cancer, pancreas cancer and adrenal cancer patients) with high sensitivity [66]. Another study by Tewarson *et al.* determined the serum sialylation in cancer patients of stomach, breast, colorectal region and gall bladder with varying degrees of metastasis before and after treatment as well as in healthy controls. Results showed that serum TSA and TSA/TP levels were significantly elevated in all cases of cancer which associated with the degree of metastasis. The disease-associated elevation of TSA/TP reversed to a certain extent after effective therapy [67]. The prognostic value of serum TSA or LSA in stomach cancer [68], thyroid cancer [69], colorectal cancer [70, 71] breast cancer [72] and malignant melanoma [73–75] was also confirmed by other groups.

Importantly, as the elevation of serum/plasma TSA and TSA/TP was observed for several cancers, it seems more promising as a prognostic and therapy efficiency marker where the requirement for cancer specificity is less than for diagnosis. Nevertheless, determining the sialic acid content in addition to more cancer-specific markers may enhance the performance of current markers. With regard to cancer specificity, Plucinsky *et al.* investigated serum TSA in cancer patients with various primary sites (rectal, melanomas, breast, gastrointestinal and pancreatic), nonmalignant diseases (villous adenomas, ulcerative colitis, hernias, endocrine disease, intestinal disease, liver disease, breast disease and regional enteritis) and healthy controls. Data analysis indicated significant increases in the average serum TSA levels in cancer and benign diseases in comparison with healthy controls. In the groups of cancer, rectal cancer showed the lowest and pancreatic showed the highest average levels of serum TSA. In the group of the benign diseases, villous adenoma patients displayed the lowest and regional enteritis recorded the highest mean value of serum TSA [76].

With regard to cancer marker potential, serum free sialic acid (FSA) as well as tissue TSA did not appear to be as conclusive as serum bound sialic acids/TSA in several types of cancer such as laryngeal cancer [30], endometrial cancer [77] and colorectal cancer [63]. In addition, serum bound sialic acid contents showed no correlation to tissue sialic acid levels in colon cancer [63]. Specifically, studies of Kim and coworkers as well as Dall’Olio *et al.* reported decreased TSA/TP content in human colonic tumors compared to the level in normal tissue from the same patients [78–80].

In contrast to this, some studies reported conflicting results. Romppanen *et al.* revealed that the elevation of serum TSA, TSA/TP and LSA concentrations in breast cancer had low sensitivities and low accuracy in differentiating between breast cancer and benign breast disease since both pathologies caused an increase of serum TSA, TSA/TP and LSA [81]. Another study by Vivas *et al.* also indicated that serum TSA or LSA seem to have little value for the early detection of cervical cancer or clinical staging (sensitivity for stage IB 0% for TSA, 27% for LSA) as serum TSA and LSA concentrations in patients with cervical cancer were only found to correlate with advanced-stage disease [82]. In addition, serum LSA/TSA measurement was found not to be useful for detecting early-stage colorectal cancer (Dukes A and B) [83], while TSA normalized to TP (TSA/TP) showed potential in early detection of colorectal cancer and follow-up of patients during treatment [71].

One may conclude from the aforementioned studies that overall sialic acid levels in serum or plasma as markers appear to show good sensitivity for various types of cancer. However, overall serum sialic acid levels are also elevated in some benign and inflammatory conditions which illustrates some lack of cancer-specificity, thereby limiting their use for early detection and cancer screening. Furthermore, there is an insufficient sample size (size range: 2–1280, median: 121, interquartile range: 73–289) of some studies and only few validation studies have been performed in an independent validation cohort from the studies mentioned above (Table 1). These may be some of the reasons why the interest in exploring overall serum sialic acid levels as a cancer marker has failed to develop further. Overall serum sialic acid measurements might, however, warrant further evaluation in combination with the measurement of existing markers for improved performance in cancer diagnosis, cancer staging, and monitoring of therapeutic response in several types of cancer.

## Protein-specific sialylation changes as serum markers in specific cancer

Studies on the utility of overall serum sialic acid levels as a cancer marker have indicated the importance of identifying cancer-specific markers, especially with regard to diagnosis. The concern over disease-specificity may be addressed through the analysis of individual sialylated glycoproteins, as reviewed in the following.

It is well known that the oncogenic process results in significant alterations of the cellular glycosylation pattern. Glycoproteins secreted by tumors may reflect the altered glycosylation machinery of cancer cells and can be detected in physiological fluids [84]. Potential tumor biomarkers may be identified based on both changes of the protein glycosylation and protein concentrations [85, 86]. In prostate cancer, determining the concentration of prostate specific antigen (PSA) alone has displayed limitations in early detection [87].PSA-specific glycosylation changes in serum from prostate cancer patients compared with controls have been characterized by employing matrix-assisted laser desorption/ionization-time-of-flight-mass spectrometry (MALDI-TOF-MS); the levels of α2–3-linked sialic acids on PSA illustrated great potential in discriminating malignant from benign conditions, thereby improving prostate cancer diagnosis [88]. Recently, Pihikova *et al.* analyzed prostate cancer serum samples applying a new electrochemical label-free method [89]. *Maackia amurensis agglutinin* (MAA, a lectin recognizing α2–3-terminal sialic acids) binding to serum PSA was significantly higher (5.3-fold) for prostate cancer samples than for healthy controls, suggesting that a combined analysis of serum PSA levels and glycoprofiling of PSA has a potential for improved detection of prostate cancer [89]. Accordingly, Llop *et al.* also evaluated the portion of α2–3-sialylated PSA in serum with a lectin immunoaffinity column and revealed that α2–3-sialic acid on PSA exhibited high performance in discriminating between high-risk prostate cancer patients and the benign prostate hyperplasia individuals [90]. In addition, the differentiation between aggressive and non-aggressive prostate cancer based on PSA α2–3-sialylation showed high sensitivity, specificity and accuracy, which indicated that α2–3-sialic acid content on PSA can improve prostate cancer diagnosis and clinical decision making [90]. Similarly, Yoneyama *et al.* measured serum α2–3-sialylated PSA using a magnetic microbead-based immunoassay in a training (*n* = 100) and a validation set (*n* = 314) of prostate cancer samples and suggested that α2–3-sialylated PSA may improve the accuracy of prostate cancer early detection [91]. With regard to other cancer types, altered glycosylation of serum MUC1, a highly sialylated glycoprotein, exhibited potential for the early diagnosis of breast cancer [92] and sialylation of serum MUC1 was also enhanced in colorectal cancer patients [93].

Another change in the protein metabolism of cancer cells is the elevated hepatic production of acute phase proteins which may play an important role in cancer pathologies. Wu *et al.* identified and validated differentially expressed sialoglycoproteins in the serum of ovarian cancer patients using a lectin-based ELISA assay and quantitative glycoproteomics analysis in three serum sample sets including one discovery set (*n* = 34) and two validation sets (validation 1: *n* = 83; validation 2: *n* = 88) and found that the sialoglycoproteins clusterin (CLUS), leucine-rich alpha-2-glycoprotein (LRG1), hemopexin (HEMO), vitamin D-binding protein (VDB), and complement factor H (CFH) were differentially expressed in the serum of ovarian cancer patients compared to benign diseases. Moreover, the decreased sialylation levels of CLUS, CFH, and HEMO in serum of ovarian cancer patients were validated which showed that these biomarkers have potential utility for diagnosis of ovarian cancer with high accuracy [94]. Kontro *et al.* found that pancreatic cancer and acute pancreatitis were related to changes of serum concentrations of sialylated glycoproteins derived from acute phase proteins and immunoglobulins by ultra-high-performance liquid chromatography (UPLC)-mass spectrometry. Pancreatitis patients showed 38 changes of site-specific glycoforms of sialylated serum glycoproteins as compared to healthy controls, whilst in pancreatic cancer patients 13 such changes as compared to healthy controls were observed, showing the potential of these glycoform changes for pancreatic cancer detection [95]. Zhao *et al.* developed a strategy for evaluating sialylated glycoprotein markers in human cancer by combining lectin assays with mass spectrometric analysis. Employing this method, approximately 130 sialylated glycoproteins were identified and sialylated plasma protease C1 inhibitor was found to be down-regulated in pancreatic cancer serum, which may serve as a marker for cancer [96]. Another study in small cell and non-small cell lung cancer patients showed different glycosylation profiles including α2–3-sialylation and expression of sialyl Lewis X (SLX) epitopes on acute phase proteins of α-1-acid glycoprotein and haptoglobin [97]*.*

Recently, emerging evidence indicates that altered immunoglobulin G (IgG) glycosylation is associated with various diseases including cancer [98, 99]. There has been an increasing interest in the analysis serum IgG glycoforms [100]. Decreased IgG sialylation levels, which associated with cancer pathogenesis or poorer prognosis, were observed in different types of cancer such as colorectal cancer [101, 102], gastric cancer [99, 103] and ovarian cancer [104]. In contrast, increased IgG sialylation was found to be linked to higher risk of multiple myeloma [105], which indicated different mechanisms involved in tumor pathologies leading to cancer-type-specific IgG sialylation changes. Decreased IgG sialylation was also described as a pro-inflammatory signal [106, 107]. The addition of sialic acid to IgG glycans has been shown to convert IgG from a pro-inflammatory to an anti-inflammatory status and, consequently, sialic acids on IgG are believed to be essential for the anti-inflammatory effect in intravenous immunoglobulin therapy [108].

Evaluating glycosylation changes on specific glycoproteins seems to be one of the most promising approaches to identify cancer-specific markers. Nevertheless, though the aforementioned results showed great potential for sialylation changes on specific glycoproteins to serve as novel or improved markers, only a few studies mentioned above were validated. Larger studies are still required for the translation of these markers from the lab to the clinics.

## Utility of serum/plasma sialyl Lewis A (CA19–9), sialyl Lewis X (SLX) and sialyl Tn (STN) antigens as markers in cancer

**Fig. 3:**
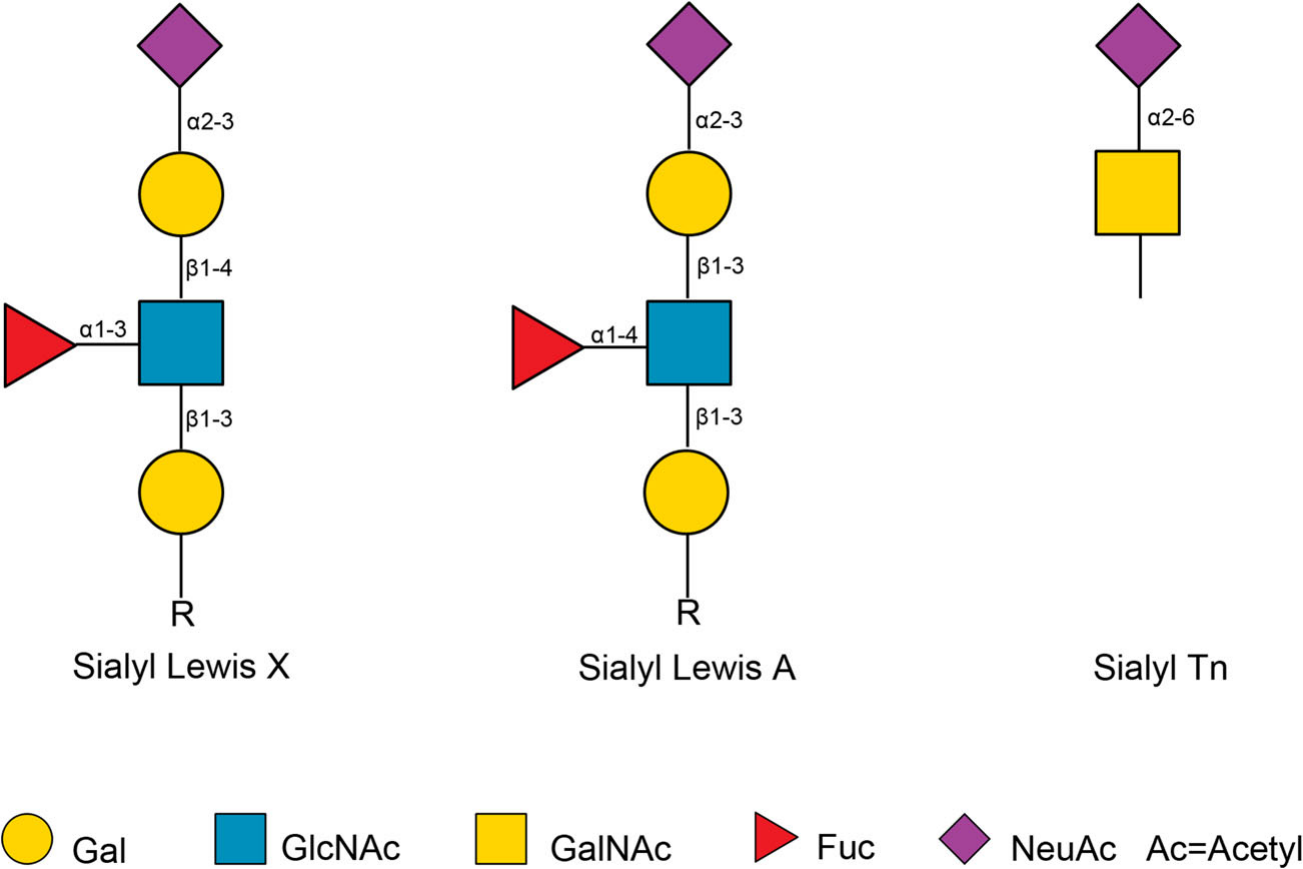
Schematic overview of sialyl Lewis A, sialyl Lewis X and sialyl Tn

Many tumor associated antigens which can be detected by available monoclonal antibodies are glycoconjugates on the cell surface [109]. Of the most common tumor-associated carbohydrate structures, sialyl Lewis A (CA19–9) and sialyl Lewis X (SLX) antigens are examples of type 1 and type 2 terminal carbohydrate structures, respectively, and sialyl Tn antigen (STN) is an example of an O-glycan core structure (Fig. 3). While CA19–9 and SLX both play an important role in cancer metastasis as ligands for endothelial cell E-selectin responsible for cell adhesion [110–114], they are also found on proteins in serum of cancer patients. Serum CA19–9, for example, showed great value as marker in the management of pancreatic cancer and is currently used in the clinic [115]. Furthermore, it displayed great potential as a diagnostic marker and predictor for metastasis in colorectal cancer [116]. High preoperative serum levels of CA19–9 and SLX have been shown to be predictive for poor prognosis of colorectal cancer after surgery [117–123]. Moreover, high preoperative serum levels of CA19–9, SLX and STN were found to be associated with liver metastasis in gastric cancer [124]. Besides this, the determination of preoperative serum STN level may also be a useful tool as a predictor of distant metastasis, mucinous carcinoma, and subsequent outcome after surgery in colorectal cancer [125]. Importantly, STN is involved in colon carcinogenesis and it is detectable in premalignant lesions of colon, and is therefore potentially suited for early detection [126, 127]. Interestingly, the highest levels of SLX is exhibited by three of the most devastating cancers: pancreatic, lung, and gastric cancer [128]. It is reported that especially SLX-containing triantennary *N-*glycans are increased in the serum of breast, prostate, ovarian, pancreatic, melanoma, peritoneal, and endometrial cancer patients [128]. In line with this, by employing sensitive HPLC-based high-throughput technology, Saldova *et al.* investigated the glycosylation of serum acute-phase glycoproteins haptoglobin, α-1-acid glycoprotein, and α1-antichymotrypsin which often carry such triantennary *N-*glycans and contained both elevated levels of SLX as well as core fucosylated agalactosylated diantennary glycans in ovarian cancer patients compared to controls. Furthermore, SLX levels combined with core fucosylated, agalactosylated diantennary glycans could significantly improve the discrimination of benign disease from ovarian cancer and showed a potential use as complementary markers for CA125 in ovarian cancer diagnosis [20]. Balmaña *et al.* identified that ceruloplasmin, one of the proteins synthetized in the liver, expressed increased SLX levels in the serum of pancreatic cancer patients and suggested the ratio of SLX/ceruloplasmin as useful biomarker for pancreatic cancer [23]. In addition, Tang *et al.* employed a new method called motif profiling and found that SLX combined with CA19–9 has potential use in the diagnosis of pancreatic cancer [129].

In summary, serum sialylation changes of glyco-antigens containing sialic acids showed great value as cancer biomarkers for improved diagnosis as well as prognosis and patient stratification in many types of cancer. As previously described, however, proper validation studies are required and a combination of glycan-epitope alterations together with other markers such as protein concentrations appears to be the most promising approach for high sensitivity and specificity.

## *N-*glycolylneuraminic acids and sialic acid modifications

Sialic acids present on cells of mammals are primarily composed of Neu5Ac and Neu5Gc, the two prevalent derivatives of sialic acids. As previously mentioned, humans are unable to synthesize Neu5Gc [26, 27]. Despite this fact, Neu5Gc was found on human epithelial and endothelial cell surfaces as a result of incorporation from dietary sources [130]. As a foreign antigen, Neu5Gc has been reported to induce an immune response which can stimulate chronic inflammation [131]. Neu5Gc is also associated with cancer pathologies and elevated levels have been detected in various types of cancer such as colorectal cancer [132, 133], liver cancer [133, 134] and ovarian cancer [135]. Furthermore, incorporated Neu5Gc can be targeted by anti-Neu5Gc antibodies in the circulation which have been identified as potential serum cancer biomarkers in humans [136]. Neu5Gc incorporation from dietary sources and its interaction with the anti-Neu5Gc antibodies in the serum have been implicated in tumor-promoting inflammation which is one of the hallmarks of cancer [137, 138]. Recently, Neu5Gc-glycoconjugates have been investigated as promising cancer vaccines, especially targeting sialylated glycolipids [139–142].

Furthermore, *O*-acetylation is a common modification of sialic acids. Though some studies have investigated their role in cancer tissues and cells lines [143, 144], to our knowledge, nothing is known on sialic acid *O*-acetylation of glycoconjugates in serum and this needs further investigation.

## Utility of serum sialidase and sialyltransferase levels in cancer diagnosis, progression and prognosis

It has been reported that changes in the expression and activity of sialyltransferases are important switches in the sialic acid metabolism of a variety of malignant cells [145]. Abnormal levels of several glycosyltransferases have been reported to be implicated in human cancer [78, 146–152]. Sialyltransferases were found to be elevated in the serum obtained from both animals and humans which bear metastasizing tumors (primary sites of the origin include: bile duct, colon, stomach, pancreas, lung, skin, breast) [147, 153–155]. Total serum sialyltransferase levels, independent of the type, were systematically studied in a long-term follow-up (measurement of the enzyme levels at 3-month intervals after surgery between 8 and 36 months) in 135 breast cancer patients, using desialylated fetuin as an acceptor to measure the enzyme activity. Results showed that sialyltransferase activities increased with breast cancer stage, indicating that serial measurements of these enzymes could be a reliable marker for monitoring disease activity and the success or failure of therapy [156]. Raval *et al.* investigated the alterations of sialyltransferase activity, but also levels of sialic acids and sialoproteins in breast cancer. This study found that elevation of all three serum markers was positively related to tumor presence and negatively associated with response to anti-cancer treatment, making high levels of these markers at diagnosis an indicator of poor prognosis [157]. Similar observations were also made for oral cavity cancer by the same group of Raval *et al.* [158]. In accordance with this, Shah *et al.* reported that serum α2–6-sialyltransferase levels have potential utility in early detection, prognosis and treatment monitoring of oral cancer [50]. These studies suggested the potential utility of these sialylation-related markers including sialyltransferases in cancer detection, prognosis and evaluation of clinical outcome. In contrast, Silver *et al.* reported that sialyltransferase measurement is relatively insensitive and has limited use in cancer prognosis [75].

Contrary to glycosyltransferases, human sialidases catalyze the removal of sialic acid residues from glycoproteins or glycolipids and have been shown to be involved in cancer progression [159]. It has been reported that sialidases can be detected in human cells and tissues. However, only few studies have focused on their presence in human serum/plasma [160–162]. Neuraminidase (NEU)3 has been found to be markedly up-regulated in a variety of human cancers such as colon [163], renal [164], ovarian [165], and prostate [166] cancer. It contributes to the augmentation of malignant properties of cancer cells most likely by causing the disturbance of transmembrane signaling [163], but only a few studies on serum/plasma have been reported. Though for many cancers increased sialylation levels have been reported, Hata *et al.* found for the first time that NEU3 could be detected in the serum of prostate cancer patients, who showed a significant increase of NEU3 activity in the serum compared with healthy subjects [167]. In their preliminary experiment, they also found higher sialidase activities in the serum of patients with bladder, testis and renal cancer [167]. Increased serum and tissue sialidase activity, measured as the extent of de-sialylation of fetuin or other glycoconjugates, were also found by Sönmez *et al.* in breast cancer patients compared to controls [168]. Interestingly, levels of sialidase activity in serum and tissue were significantly different between Grade I–II and III breast cancer patients, indicating the potential use of sialidase in serum and tissue as cancer biomarker for evaluating cancer progression and patient stratification [168].

Though the mechanisms and function of glycan-related enzymes is still largely obscure, measuring these enzymes may turn out to be another approach in order to find potential clinical cancer markers, and warrants further in-depth examination.

## Useful techniques for the measurements of serum/plasma sialylation changes

Serum/plasma sialylation changes have been established as a potential tumor marker for patients with various cancers. A variety of methods for the detection and estimation of free sialic acids and sialic acids bound to glycoproteins or glycolipids have been performed. The earliest methods tended to be colorimetric/spectrophotometric assays including orcinol methods, resorcinol methods, periodic acid/thiobarbiturate methods, and the periodic acid/methyl-3-benzothiazolone-2-hydrazone method [169–174]. For these methods, sialic acids are first released by acid hydrolysis or neuraminidase treatment, then reacted with reagents to form a chromogen which can be extracted and measured based on their spectral absorption. Some groups have also attempted to chemically dissect serum sialic acid into sialolipid and sialoglycoprotein components in order to measure LSA [175]. While colorimetric/spectrophotometric methods are still widely used, simple to handle and relatively straightforward to be used in the clinics, interferences can lead to overestimation of the sialic acid content. Therefore, different modifications of the colorimetric/spectrophotometric protocols as well as various fluorescence assays [176], enzymatic assays [177], and chromatographic methods for sialic acid content determination have been developed in order to pursue higher sensitivity or specificity, and easy and fast procedures.

Chromatographic separation, in particular, allows the successful separation of the sialic acids from interfering compounds. Different chromatographic approaches have been published for the determination of sialic acid content including direct approaches such as Anion-Exchange Chromatography with Pulsed Amperometric Detection (HPAE-PAD) as well as gas chromatography with mass spectrometry (GC-MS), thin-layer chromatography and the highly sensitive high performance liquid chromatographic (HPLC) method [178–181]. While approaches such as HPAE-PAD measure the sialic acids directly, others utilize an indirect method through derivatization of the sialic acid followed by HPLC and photometric or fluorescence detection. Early developments explored sugar-borate complexes in combination with different ion exchange HPLC separations and photometric detection, allowing the quantification of (*N-* and O-acetylated) sialic acids without prior extensive purification [181]. One of the most common fluorescence labels for sialic acids is 1,2-diamino-4,5-methylenedioxybenzene dihydrochloride (DMB) which has been applied to detect and quantify different sialic acid variants in combination with HPLC separation and fluorescence detection as well as liquid chromatography-electrospray ionization-mass spectrometry (LC-ESI-MS) [182, 183].

Mass spectrometry as a detection method has become an important technology for glycan analysis. It offers sensitivity, high accuracy, tolerance for the sample impurity, and compatibility with various separation techniques. MALDI-TOF-MS has become a major approach for glycan profiling of human serum (in high-throughput manner) and identifying sialylated glycoproteins. However, the cleavage of the sialic acid moiety by in- and post-source decay can cause biases in the determination of sialylated glycans by MALDI-MS. Many chemical derivatization methods were introduced to stabilize the sialylated glycan during MALDI-MS analysis including permethylation [184], esterification [185, 186], amidation [187], methylamidation [188] and dimethylamidation [189] and have increased the sensitivity of detection and the stability of sialic acids.

Recently, Wang *et al.* reported on a highly specific and sensitive LC-MS/MS glycomic method which can be used to quantitatively determine the level of free and conjugated forms of sialic acids, namely *N*-acetylneuraminic acid, *N-*glycolylneuraminic acid and 2-keto-3-deoxy-D-glycero-D-galacto-nononic acid (KDN). This was applied to human cancers and a subset of matched lymph nodes [190]. This LC-MS/MS method showed higher sensitivity than the HPLC-based DMB method used in their previous studies [191]. Furthermore, selected reaction monitoring (SRM) has become a very popular MS detection mode for LC-MS/MS methods due to its high capability of selection, high sensitivity and specificity - gradually attracting more attention from researchers regarding qualitative and quantitative analysis of sialic acids [192]. Similar studies have been performed by Shi *et al.* [193] and Capote *et al.* [194] concerning sialic acid quantitation in human plasma employing LC-MS/MS and good accuracy and a low coefficient of variation were reported. Notably, when highly sensitive methods are applied, there is a risk of cross-contamination by sialic acids through sample-handling (particularly samples that contain very low levels of sialic acids) . It is recommended that measures should be taken to limit this risk of contamination [195].

## Putative mechanisms for the serum/plasma sialylation changes

Many malignant cells are characterized by increased expression of sialic acids on the cell surfaces and membranes [34]. In certain cancers, increased activity of sialyltransferase and the high turnover of tumor cells might lead to spontaneous shedding of aberrant sialic acid-containing cell surface glycoconjugates into the circulation and cause the high sialic acid concentration in the serum [196]. In one study, tumor tissue TSA/TP was found to be significantly higher than serum TSA /TP supporting the hypothesis of an enhancement in tumor sialoglycoconjugate biosynthesis and shedding being the cause of an increase in serum sialic acid content [197]. A decrease in serum sialoglycoconjugate degradation and/or an altered clearing of these glycoconjugates by the liver could also account for the elevation of serum sialic acids [63]. The increased serum sialic acid concentration was directly correlated to an increase in the concentration and the degree of sialylation of tumor secreted products such as alkaline phosphatase, MUC5AC mucin and CA19–9 which are sialoglycoconjugates, as reported by Wongkham *et al.* [197]. It is speculated that serum MU5AC mucin and antigen CA19–9 are related to the increased amount of white blood cells as well as secretion of cytokines and glycoproteins from immune cells in serum of cancer patients; one possible explanation for the elevation of serum TSA [197]. On the other hand, increased activity of serum sialyltransferases has been shown in several cancers [63, 198, 199] which may reflect an inflammation reaction to the tumor, resulting in more sialic acid-rich glycoprotein synthesis and release from liver [200].

Altered glycosylation regulated by epigenetics such as histone modification, DNA methylation or remodeling of microRNA may be another concept for inducing increased sialylation in cancer which would suggest a systematic transformation from healthy to diseased conditions [201]. For example, increased levels of branching and sialylation of *N-*glycans after 5-AZA-2′-deoxycytidine treatment in the ovarian cancer cell line OVCAR3 and increased production of the SLX epitope on the MUC1 protein in HCT15 colon cancer cells were found by Saldova *et al.* [202] and Chachadi *et al.* [203], respectively.

In addition, higher serum TSA may be related to different concentrations and glycosylation patterns of acute-phase proteins. Accordingly, Crook *et al.* found significant correlation between serum TSA and serum levels of some acute phase proteins such as α1-antichymotrypsin, α-1-acid glycoprotein and serum C-reactive protein [204]. Taniuchi *et al.* reported that serum TSA correlates well with the acute-phase protein response and in particular with serum concentrations of α-1-acid glycoprotein [205]. Being an acute phase protein, α-1-acid glycoprotein is synthesized by the liver and secreted into the circulation. Its serum concentration rises in response to inflammatory stimuli, potentially increasing the concentration two- to four-fold [206]. During the early stages of an acute-phase immune response, the SLX levels of α-1-acid glycoprotein increase significantly, which continues throughout the whole acute phase immune response [206–208].

The involvement of acute-phase proteins in these clinical conditions forms the most plausible explanation for the elevated serum TSA levels, raising the question of the relationship between cancer and inflammation. On the other hand, studies focusing on liver dysfunction showed a decreased serum level of mucoproteins in liver cirrhosis, which was in opposition to the results in neoplastic transformation [200]. In chronic liver diseases the mean sialic acid level was lower than in a group of non-inflammatory and non-neoplastic diseases [200]. The α-1-acid glycoprotein is markedly affected by a disturbance of synthesis of serum glycoproteins due to chronic liver insufficiency as the liver is the main site of synthesis for these glycoproteins [200]. Another study confirmed the conclusions showing that in cases of liver cirrhosis the TSA concentration is near normal [209]. The explanation is not so clear but may reflect heterogeneity of levels of acute phase proteins or differing degrees of glycoprotein sialylation in these individuals. With regard to the aforementioned different types of cancer one would anticipate an acute phase response, though this does not account for the entire serum sialic acid elevation since sialylated glycolipids, so-called gangliosides, also contribute slightly to the serum TSA content (1–2%) [210].

The potential mechanisms underlying the sialylation-related changes in cancer discussed here also illustrated the complexity and limitations in using altered sialic acids as tumor markers as it has an acute phase reactant and most of the proteins that increase in acute phase are sialylated (dominated by α2–3-sialylation on these proteins in the form of SLX). Therefore, most investigators indicated that serum sialic acids could be more useful in monitoring of cancer patients and follow up after therapy rather than early-detection.

## Conclusion and future perspectives

Serum/plasma sialylation changes in cancer patients have been studied to evaluate their potential as tumor marker. Sialylation changes in serum/plasma can be detected during cancer initiation, progression and treatment which show potential in a variety of clinical applications. Summarizing the applications of serum sialylation-related markers discussed above, the majority of cancers showed alterations in total sialic acid levels, concentrations and degrees of sialylation of specific glycoproteins, levels of specific sialo-glycan antigens and/or activity of sialylation-related enzymes, *i.e.* sialidases and sialyltransferases (see also Tables 1, 2, 3, 4 for a comprehensive overview). Various investigators have suggested that overall serum sialic acid concentration may be a valuable biochemical marker in detecting metastases, stages of a disease, risk for recurrence and evaluating therapeutic response, potentially in combination with other markers to increase the cancer type-specificity. Sialidases, which catalyze the removal of sialic acid residues from glycoproteins and glycolipids, and sialyltransferases, which are involved in the formation of sialylated glycans, were also found to be differentially expressed in the serum, providing likewise potential cancer biomarkers, but studies hitherto have displayed some limitations. Particularly worth mentioning are alterations in sialylation of individual glycoproteins which can help to improve the specificity of sialylation-related markers. For example, increased α2–3-linked sialylation of PSA is reported to have potential as biomarker for prostate cancer. Additionally, increased IgG sialylation is useful for the assessment of risk of multiple myeloma, while decreased IgG sialylation is associated with poor prognosis in colorectal cancer. Furthermore, sialylation changes on acute-phase proteins as well as alterations of glyco-antigens such as CA19–9 and SLX in serum showed value for diagnosis as well as prognosis and patient stratification.

**Table 1:** Summary of serum overall sialylation changes (TSA, LSA, bound sialic acid, TSA/TP and bound sialic acid/TP) in various cancers. The columns include (1) cancer types, (2) cohort size, (3) methods used for the detection of sialylation changes, (4) major findings for each study, (5) the trend of the sialylation changes and (6) the references for each study; FSA = free sialic acid; HPLC = high performance liquid chromatography; LSA = lipid-bound sialic acid; TP = total protein; TSA = total sialic acid

Malignancy	Cohort Size	Methods	Major findings	Effect in the cancer	Ref No.
Laryngeal cancer	Laryngeal cancer(*n* = 35); Healthy controls (*n* = 34)	Serum TSA and free sialic acid: thiobarbituric acid method; Serum bound sialic acid: determined as the difference between TSA and FSA; α-1-acid glycoprotein: nephelometric method	Higher levels of serum bound sialic acid and α-1-acid glycoprotein, but not free sialic acid, have correlation with the stage of the cancer	Serum sialic acid and α-1-acid glycoprotein increased in laryngeal cancer	30
Oral cancer	Oral cancer (*n* = 130); Precancerous conditions (*n* = 75); Healthy controls (n = 100)	Serum and tissue TSA: spectrophotometric method; sialyltransferase activity and sialoproteins: linkage-specific lectins	Usefulness of serum and tissue TSA and linkage-specific sialoproteins and sialyltransferase as biomarker in early detection, prognostication and treatment monitoring of oral cancer	Serum and tissue sialic acid and linkage-specific sialoproteins and sialyltransferase increased in oral cancer	50
Oral pre-cancer	Oral cancer (*n* = 25); Precancerous conditions (*n* = 50); Healthy controls (*n* = 25)	Serum TSA and LSA: spectrophotometric method	Serum TSA and LSA positively correlated with grades of dysplasia of oral pre-cancer and cancer; LSA showed great potential of clinical utility in indicating premalignant change	Serum TSA and LSA increased in oral pre-cancer and cancer	51
Oral pre-cancer	Oral cancer (*n* = 30); Precancerous conditions (*n* = 30); Healthy controls (n = 30)	Serum TSA: resorcinol reagent method	Usefulness of serum TSA in monitoring early changes of oral cancer; Positive correlation of serum TSA with stage and tumor burden	Serum TSA increased in oral pre-cancer and cancer	52
Oral pre-cancer	Oral cancer (n = 25); Precancerous conditions (n = 25); Healthy controls (n = 25)	Serum TSA: spectrophotometric method	Increased serum TSA has potential utility in initial diagnosis of leukoplakia and squamous cell oral cancer	Serum TSA increased in oral pre-cancer and cancer	53
Oral pre-cancer	Oral cancer (n = 25); Precancerous conditions (n = 25); Healthy controls (n = 25)	Serum TSA: spectrophotometric method	Serum TSA has potential utility in early detection of oral cancer	Serum TSA increased in oral pre-cancer and cancer	54
Oral pre-cancer	Oral cancer (n = 100); Precancerous conditions (n = 50); Healthy controls (*n* = 100)	Serum TSA: spectrophotometric method	Serum and salivary TSA/TP showed usefulness in monitoring early changes during oral cancer transformation	Serum TSA/TP increased in oral pre-cancer and cancer	55
Oral pre-cancer	Oral cancer (*n* = 41); Precancerous conditions (*n* = 20); Healthy controls (*n* = 20)	Serum TSA and LSA: spectrophotometric method	Potential utility of serum TSA and LSA in oral cancer diagnosis; Serum TSA and LSA positively correlated with clinical stage of the malignancy	Serum TSA and LSA increased in oral pre-cancer and cancer	56
Oral pre-cancer	Oral leukoplakia (n = 30); Healthy controls (*n* = 30)	Serum TSA and LSA: spectrophotometric method	Grades of epithelial dysplasia of oral leukoplakia positively correlated with serum TSA levels, which can serve as markers for the malignant transformation in oral leukoplakia	Serum TSA and LSA increased in oral pre-cancer	57
Cholangiocarcinoma	Cholangiocarcinoma (*n* = 89); Benign hepatobiliary diseases (*n* = 38); Healthy controls (*n* = 43)	Serum TSA: spectrophotometric method	Serum TSA has high adjunct diagnostic values for discriminating cholangiocarcinoma, benign hepatobiliary diseases and healthy controls	Serum TSA have an increasing trend from controls, benign to cancer	59
Cervical cancer	Cervical cancer (*n* = 108); Healthy controls (*n* = 125)	Serum TSA and LSA: spectrophotometric method	Serum TSA and LSA have diagnostic and treatment monitoring value in cervical cancer	Serum TSA and LSA increased in cervical cancer	60
Leukemia	Leukemia patients (*n* = 145); Anemia patients (*n* = 77); Healthy controls (*n* = 150)	Serum TSA/TP and LSA: spectrophotometric method	Usefulness of evaluated serum TSA/TP and LSA are useful in diagnosis and treatment monitoring of leukemia	Serum TSA/TP and LSA increased in leukemia patients and anemia patients	61
Colorectal cancer	Patients (*n* = 177; 109 patients with colon and 68 patients with rectal); Healthy controls (n = 50)	Serum TSA and LSA: spectrophotometric method	Serum TSA is sensitive marker and has potential utility in the earliest diagnosis of colorectal, it also play important roles in cancer progression	Serum TSA, but not LSA, significantly increased in the cancer group	62
Lung cancer	Lung cancer (n = 12); Chronic obstructive lung disease (n = 6); Controls (*n* = 64; no neoplastic disease)	Serum sialic acid: thiobarbituric acid methods	Elevated serum sialic acid showed usefulness as a cancer biomarker in lung cancer	Serum sialic acid elevated in lung cancer	65
Colorectal cancer	Colorectal cancer (n = 30); Healthy controls (*n* = 810)	TSA and free sialic acid: thiobarbituric acid method; Bound sialic acid: determined as the difference between TSA and FSA	Serum TSA/TP and bound sialic acid/TP have positive correlation with tumor stage; Serum and tissue bound sialic acid have no correlation	Serum TSA, bound sialic acid,TSA/TP and bound sialic acid/TP were significantly higher in cancer; tissue TSA/TP and bound sialic acid/TP were significantly decreased	63
Breast cancer	Breast cancer (*n* = 65); Controls (*n* = 56)	Serum sialic acid: spectrophotometric method	Serum sialic acid correlated with tumor stage; Serum sialic acid have no correlation with CEA values	Serum sialic acid elevated in breast cancer	64
Endometrial cancer	Cancer (*n* = 52); Healthy controls (n = 20)	Serum and tissue sialic acid: spectrophotometric method	Serum TSA positively correlated with tumor stages; Tissue sialic acid had no correlation with cancer stages	Serum TSA significantly increased in cancer	77
Several types of cancer	Breast cancer (*N* = 54); Lung cancer (n = 17); Colon cancer (n = 15); Ovarian cancer (n = 7); Prostate cancer (n = 5); Leukemia (n = 4); Gastrointestinal cancer (n = 4); Thyroid cancer (n = 3); Pancreatic cancer (n = 3); Adrenal cancer (n = 2); Patients with non-malignant diseases (*n* = 16); Healthy controls (*n* = 50)	Plasma LSA: spectrophotometric method	Plasma LSA showed potential utility as a prognostic marker in a variety of neoplastic conditions with high sensitivity	Plasma LSA elevated in a variety of types of cancer	66
Several types of cancer	Healthy controls (n = 30); Cancer (*n* = 78; including patients of stomach, breast, colorectal region and gall bladder cancer)	Serum TSA and LSA: spectrophotometric method	Serum TSA, TSA/TP positively correlated with metastasis; TSA and TSA/TP are sensitive markers for detection of malignancy and evaluating the efficacy of therapies	Serum TSA and TSA/TP significantly raised and serum TP decreased in these types of cancer	67
Stomach cancer	Cancer (*n* = 48); Healthy controls (n = 20)	Serum TSA and LSA: spectrophotometric method	Serum TSA and LSA showed potentials as indicators of poor or good prognosis	Serum TSA and LSA of cancer before therapy were higher than control and that after receiving therapy	68
Thyroid cancer	First part: Cancer (n = 50); Healthy controls (n = 20); Second part: Cancer (*n* = 11); Adenomatous hyperplasia(n = 8; as control)	Serum and tissue TSA: thiobarbituric acid methods	Usefulness of sialic acid in follow-up and therapeutic response evaluation	Serum and tissue sialic acid levels in various types of thyroid cancer were significantly higher than in controls	69
Colorectal cancer	Colorectal cancer (*n* = 97); Acute and chronic disorders (*n* = 69); Benign colorectal polyps (n = 17); Healthy controls (*n* = 195)	Serum TSA: HPLC; LSA: resorcinol procedure	Serum TSA and LSA correlated with the extent of metastasis; TSA and LSA had strong correlation; TSA and LSA showed potential as supplemental markers for staging and monitoring cancer	Serum TSA elevated in 32% nonmalignant disorders, 28% localized cancer, and 87% of metastatic cancer	70
Colorectal cancer	Colorectal cancer (*n* = 146); gastrointestinal disease (*n* = 73); Normal controls (*n* = 96)	Serum TSA: spectrophotometric method	Usefulness of serum TSA/TP in colorectal cancer monitoring	Serum TSA/TP have an increasing trend from normal controls, pathologic controls to cancer	71
Breast cancer	Primary operable breast cancer (n = 64); Recurrent metastatic breast cancer (*n* = 61); Benign breast disease (*n* = 106); Normal controls (n = 78)	Serum LSA: spectrophotometric method	Usefulness of serum LSA in evaluating disease progression, prognosis and identifying resistance to therapy	LSA levels greater than cutoff were not seen in normal subjects; presented in 13% benign breast disease, 47% primary breast cancer and 62% recurrent metastatic breast cancer	72
Malignant melanoma	Melanoma (n = 50); Healthy controls (*n* = 40)	Serum TSA: enzymatic method	Serum TSA can discriminate cancer and healthy controls; More useful for staging and prognosis	Serum TSA increased in cancer	73
Malignant melanoma	Melanoma (n = 25); Healthy controls (n = 30)	Serum sialic acid: thiobarbituric acid method	Increased serum sialic acid correlated with tumor burden due to therapy or recurrence	Serum sialic acids were significantly elevated in the melanoma	74
Malignant melanoma	Melanoma (*n* = 66); Healthy controls (n = 66); Rheumatoid arthritis (n = 20)	Serum-bound sialic acid: thiobarbituric acid method; Serum sialyltransferase: cytidine 5′-monophosphate-N-[4-14C]acetylneuraminic acid incorporation in desialylated fetuin	Serum sialic acid showed higher value for monitoring recurrence of cancer than sialyltransferase; Sialic acid and sialyltransferase had correlation	Serum bound sialic acid and sialyltransferase were higher in rheumatoid arthritis patients; Serum sialic acid increased in cancer of different stages	75
Cervical cancer	Cervical cancer (n = 88); Benign uterine or ovarian disease (*n* = 44); Healthy controls (*n* = 26)	Serum TSA and LSA: spectrophotometric method	Serum TSA or LSA showed no value for early detection or as complemental marker for clinical staging	Serum TSA and LSA increased in advanced stage of cancer	82

**Table 2:** Summary of sialylation changes of serum specific glycoproteins in specific cancer. The columns include (1) cancer types, (2) cohort size, (3) methods used for the detection of sialylation changes, (4) major findings for each study, (5) the trend of the sialylation changes and (6) the references for each study; ELISA = enzyme linked immunosorbent assay; PSA = prostate specific antigen

Malignancy	Cohort Size	Methods	Major findings	Effect in the cancer	Ref No.
Prostate cancer	2 cancer patients	Matrix-assisted laser desorption/ionization time-of-flight mass spectrometry (MALDI-TOF-MS)	Levels of α2–3-linked sialic acids on PSA showed great potential in discriminating malignant from benign conditions and improving prostate cancer diagnosis	\	88
Prostate cancer	\	PSA sialic acid: electrochemical label-free method	Combined analysis of serum PSA levels and glycoprofiling of PSA could improve the detection of cancer	α2–3-terminal sialic acid of PSA was significantly higher in prostate cancer	89
Prostate cancer	Cancer (n = 44); Benign prostatic hyperplasia (*n* = 29)	PSA sialic acid: lectin immunoaffinity column	α2–3-sialic acid showed great potential in discriminating between high-risk prostate cancer patients and the benign prostate hyperplasia individuals	α2–3-terminal sialic acid of PSA was higher in prostate cancer than benign disease	90
Prostate cancer	Training set: Cancer (n = 50); Non-cancer controls (n = 50) Validation set: Cancer (*n* = 138); Non-cancer controls (*n* = 176)	PSA sialic acid: magnetic microbead-based immunoassay	Usefulness of α2–3-sialylated PSA in the accuracy improvements of prostate cancer early detection	α2–3-terminal sialic acid of PSA was higher in prostate cancer than non-cancer	91
Ovarian cancer	Discovery set: Cancer (*n* = 22); Benign tumor (n = 12); Confirmation 1: Cancer (n = 50); Benign disease (*n* = 18); Healthy controls (n = 15); Confirmation 2: Cancer (n = 43); Benign disease (n = 30); Healthy controls (n = 15)	lectin-based ELISA assay and quantitative glycoproteomics analysis	Serum sialoprotein and sialylation changes of proteins have potential utility for diagnosis of ovarian cancer with high accuracy	Higher levels of sialoprotein and sialylation of proteins in cancer	94
Pancreatic cancer	Cancer (n = 10); Acute pancreatitis (N = 5); Healthy controls (n = 16)	Ultra-high-performance liquid chromatography (UPLC)-mass spectrometry (MS)	Altered sialylated glycoproteins have great potential as novel biomarker for pancreatic cancer detection	Higher levels of sialoprotein in cancer and benign than controls	95
Pancreatic cancer	Cancer (n = 3); Healthy controls (*n* = 3)	Lectin assays combining with mass spectrometric analysis	Sialoprotein and sialylation changes of the protein have usefulness as glycan related cancer biomarker	Sialylated plasma protease C1 inhibitor decreased in cancer	96
Lung cancer	Cancer (*n* = 46); Healthy controls (n = 20)	ELISA tests	Usefulness of glycosylation profiles including α2–3-sialylation and sialyl Lewis X on alpha-1-acid glycoprotein and haptoglobin as cancer biomarker	α2–3 sialylation and sialyl Lewis X were elevated in total serum of cancer	97
Gastric cancer	Cancer (*n* = 80); Healthy controls (*n* = 51)	Liquid chromatography-electron spray ionization-mass spectrometry (LC-ESI-MS)	IgG glycosylation is related to cancer pathogenesis, progression and prognosis	IgG sialylation decreased in cancer	99
Colorectal cancer	Cancer (*n* = 129)	Ultra-high-performance liquid chromatography (UPLC)	IgG glycosylation is related cancer prognosis	IgG sialylation decreased in cancer	101
Colorectal cancer	Cancer (*n* = 760); Healthy controls (*n* = 538)	Ultra-high-performance liquid chromatography (UPLC)	IgG glycosylation is related cancer prognosis	IgG sialylation decreased in cancer	102
Gastric cancer	Cancer (*n* = 403); benign disease (*n* = 443)	Matrix-assisted laser desorption/ionization-Fourier transform ion cyclotron resonance mass spectrometry (MALDI-FTICR MS)	IgG Fc glycoforms could reflect difference of pathophysiological states between gastric cancer and benign disease and also showed diagnostic capability	IgG sialylation decreased in cancer	103
Multiple myeloma	Monoclonal gammopathy of uncertain significance (n = 14); Multiple myeloma (n = 41); Solitary plasmacytoma (n = 5)	High pressure anion exchange chromatography with pulsed electrochemical detection	The ratio of neutral to sialylated glycans showed potential as a new marker for multiple myeloma	IgG sialylation increased in malignancy	105

**Table 3:** Summary of serum SLX, CA19–9 and STN changes as markers in cancers. The columns include (1) cancer types, (2) cohort size, (3) methods used for the detection of sialylation changes, (4) major findings for each study, (5) the trend of the sialylation changes and (6) the references for each study; CA19–9 = sialyl Lewis A; HPLC = high performance liquid chromatography; LC-ESI-QTOF MS = liquid chromatography-electron spray ionization- quadrupole time-of-flight mass spectrometry; MALDI-TOF-MS = matrix-assisted laser desorption/ionization time-of-flight mass spectrometry; SLX = sialyl Lewis X; STN = sialyl Tn

Malignancy	Cohort Size	Methods	Major findings	Effect in the cancer	Ref No.
Ovarian cancer	Initial pilot study: Cancer (n = 3); Healthy control (n = 5); Main part of the study: Cancer (*n* = 56); Benign disease (*n* = 27); Healthy control (n = 7)	HPLC, MALDI-TOF-MS and ESI-MS	SLX combined with fucosylated diantennary glycoforms can improve the discrimination of benign disease from ovarian cancer; Potential utility as complementary markers for CA125 in ovarian cancer diagnosis	Serum SLX increased in cancer	20
Pancreatic cancer	Cancer (n = 20); Chronic pancreatitis (n = 14); Healthy controls (n = 13)	Immunodetection and LC-ESI-QTOF MS analysis	The ratio of SLX/ceruloplasmin may serve as useful biomarker for pancreatic cancer	Ceruloplasmin(synthetized in liver) expressed increased SLX in the serum of cancer	23
Pancreatic cancer	48 plasma samples and a blinded set of 200 samples	Motif profiling	Plasma SLX combined with CA19–9 has potential use in the clinical diagnosis of pancreatic cancer	Plasma SLX was elevated in pancreatic cancers	129
Colorectal cancer	Cancer (*n* = 300)	Electrochemiluminescent assay	Usefulness of serum CA19–9 in colorectal cancer diagnosis, staging and suggesting metastasis	Serum CA19–9 increased with stages of cancer	116
Colorectal cancer	Cancer (*n* = 293)	Immunoradiometric method	Serum CA19–9 sensitivity related to cancer stage and showed great values in colorectal cancer prognosis	Serum CA19–9 increased with stages of cancer	117
Colorectal cancer	Cancer (*n* = 121; advanced stage)	Commercially available assay kit	Serum and tissue CA19–9 detection are useful in the assessment of high risk of cancer recurrence and death (prognosis values)	Positive (higher than cut-off) preoperative and postoperative serum CA19–9 were predictive of increased cancer mortality	118
Colorectal cancer	Cancer (*n* = 85; advanced stage)	Assay kit	Serum CA19–9 have great values as colorectal cancer prognostic indicator	Serum CA19–9 negatively correlated with survival	119
Colorectal cancer	Cancer (*n* = 206)	Commercially available radioimmunoassay kit	Serum CA19–9 is useful as preoperative indicator of metastasis and prognosis	Higher serum CA19–9 associated with metastasis and poorer prognosis	120
Colorectal cancer	Cancer (n = 78; advanced stage)	Assay kit	STN expression are important prognostic factors in patients with advanced colorectal cancer	STN in serum had the strong association with survival	121
Colorectal cancer	Cancer (*n* = 117)	Radioimmunoassay	Combined assay of serum carcinoembryonic antigen, CA19–9, STN and SLX improved colorectal cancer diagnosis and follow-up	Increased marker values correlated to poorer prognosis	122
Colorectal cancer	Cancer (*n* = 284)	Assay kit	Serum initial CA19–9 could be independent prognostic biomarkers in metastatic colorectal cancer	Elevated serum CA19–9 was unfavorable prognostic factors.	123
Colorectal cancer	Cancer (*n* = 308)	Commercially available radioimmunoassay kit	High serum CA19–9, SLX, and STN were strongly associated with distant metastasis and also showed prognosis values	High serum levels related to distant metastasis	125
Gastric cancer	Cancer (*n* = 180)	Assay kit	High serum CA19–9, SLX and STN were associated with liver metastasis; High serum SLX and STN were related to peritoneal dissemination; High serum CA19–9 has potential as an independent predictor for lymph node metastasis	Serum levels of these markers increased in metastasis	124

**Table 4:** Summary of serum sialidase and sialyltransferase changes in cancer. The columns include (1) cancer types, (2) cohort size, (3) methods used for the detection of sialylation changes, (4) major findings for each study, (5) the trend of the sialylation changes and (6) the references for each study

Malignancy	Cohort Size	Methods	Major findings	Effect in the cancer	Ref No.
Breast cancer	Cancer (*n* = 135)	De-sialylated fetuin as acceptor to measure the enzyme activity	Serum sialyltransferase could be a reliable marker for the monitoring of disease activity and success or failure of therapy	Serum sialyltransferase increased with higher breast cancer activity	156
Breast cancer	Cancer (*n* = 225); Benign breast disease (n = 100); Healthy controls (n = 100)	Sialyltransferase: de-sialylated fetuin as acceptor; total sialic acids and free sialic acid: spectrophotometric method	Serum sialic acid forms and sialyltransferase are of clinical value in monitoring clinical course and in assisting the diagnosis of breast cancer.	Increased serum sialic acid and sialyltransferase were positively associated with presence of malignant tumor and negatively with response to anticancer treatment; Malignant tissues showed elevated sialic acid and sialyltransferase	157
Oral cancer	Cancer (*n* = 210); Precancerous conditions (n = 100); Healthy controls (*n* = 100); Cancer follow-up (*n* = 394;after treatment)	Serum sialic acid: spectrophotometric method; Sialyltransferase: radioassay; α2–6 sialoproteins: lectin affinity chromatography.	Potential utility of serum sialic acid and sialyltransferase in prognostication and treatment monitoring of oral cancer; α2–6 sialylated proteins were associated with changes of serum sialic acid and sialyltransferase	Serum sialic acid and sialyltransferase were elevated in oral cancer	158
Prostate cancer	Cancer (n = 34); Healthy controls (n = 13);	Sialidase assays: fluorometric method	Serum sialidase (NEU3) has potential utility as novel diagnostic cancer marker	Serum sialidase activity significantly increased in prostate cancer compared with healthy	167
Breast cancer	Cancer (n = 26); Healthy controls (n = 31);	Sialidase: measured as extent of de-sialylation of fetuin or other glycoconjugates	Serum and tissue sialidase in breast cancer significantly increased compared with in the controls; There also existed significant difference between the levels of serum and tissue sialidase	Serum and tissue sialidase in breast cancer significantly increased compared with in the controls	168

As serum/plasma sialylation changes have been established as a potential tumor marker for patients with various cancers, more accurate methods with high sensitivity, high specificity and less time-consuming sample preparations are needed for the detection and evaluation of free and glycosidically-bound sialic acids. A variety of reports on the improvements of the methods have been published and mass spectrometry-based methods are gaining more interest.

Moreover, the mechanism behind the sialylation-related changes in different cancers remain poorly understood, though several possibilities of the increase in serum sialic acid are currently being considered: an intensified release of sialic acid-containing cell surface glycoconjugates from tumor cells, an increased concentration and/or glycosylation of normal serum glycoproteins, secondary inflammatory reactions leading to an output of acute phase proteins from the liver, or increased sialylation of serum glycoproteins resulting from epigenetic regulation.

Overall, serum sialylation changes are prominent in various cancers and offer a broad range of opportunities for novel markers. Future research should focus on defining these sialylation changes in a more specific manner revealing protein-specific changes and, importantly, validation studies with larger sample cohorts are needed to confirm and eliminate current markers. Likewise, mechanistic insights are needed in order to shed light on the potential interplay of glycomic changes in the malignant tissue, the liver and plasma cells, and the role of sialylation changes inflicted post-secretion by sialidases and transferases.
